# Neuroprotective Effect of Clemastine Improved Oligodendrocyte Proliferation through the MAPK/ERK Pathway in a Neonatal Hypoxia Ischemia Rat Model

**DOI:** 10.3390/ijms25158204

**Published:** 2024-07-27

**Authors:** Maria E. Bernis, Charlotte Hakvoort, Efe Nacarkucuk, Hannah Burkard, Anna-Sophie Bremer, Margit Zweyer, Elke Maes, Kora A. Grzelak, Hemmen Sabir

**Affiliations:** 1 Neonatologie und Pädiatrische Intensivmedizin, Eltern-Kind-Zentrum, Universitätsklinikum Bonn, 53127 Bonn, Germany; maria.bernis@dzne.de (M.E.B.); hakvoort.charlotte@gmail.com (C.H.); efe.nacarkucuk@dzne.de (E.N.); hannah.burkard@dzne.de (H.B.); anna.bremer@dzne.de (A.-S.B.); margit.zweyer@dzne.de (M.Z.); elke.maes@dzne.de (E.M.); koraalexandra.grzelak@dzne.de (K.A.G.); 2 Deutsches Zentrum für Neurodegenerative Erkrankungen (DZNE), 53127 Bonn, Germany

**Keywords:** hypoxia, ischemia, white matter, injury, oligodendrogenesis, clemastine, neuroprotective

## Abstract

Neonatal hypoxic-ischemic encephalopathy is the most common cause of long-term disability in term neonates, and white matter injury is the primary cause of cerebral palsy. Therapies that focus on the neuroprotection of myelination and oligodendrocyte proliferation could potentially ameliorate long-lasting neurological impairments after hypoxic-ischemic encephalopathy. Clemastine, a histamine H1 antagonist, has been shown to exert neuroprotective effects in multiple sclerosis and spinal cord injury by promoting oligodendrogenesis and re-myelination. In this study, we demonstrated the neuroprotective effects of clemastine in our rat model of neonatal hypoxic-ischemic brain injury. Animals received a single intraperitoneal injection of either vehicle or clemastine (10 mg/kg) for 6 consecutive days. Our results showed a significant reduction in white matter loss after treatment, with a clear effect of clemastine on oligodendrocytes, showing a significant increase in the number of Olig2+ cells. We characterized the MAPK/ERK pathway as a potential mechanistic pathway underlying the neuroprotective effects of clemastine. Altogether, our results demonstrate that clemastine is a potential compound for the treatment of hypoxic-ischemic encephalopathy, with a clear neuroprotective effect on white matter injury by promoting oligodendrogenesis.

## 1. Introduction

Neonatal hypoxic-ischemic encephalopathy (HIE) is the most common cause of death and long-term disabilities in term neonates [1]. Hypoxia-ischemia (HI) causes inflammation, oxidative stress, neuronal death, and white matter injury [2]. Therapeutic hypothermia (TH) is the unique standard neuroprotective treatment that is currently available to counteract the progression of HI injury in near-term and term newborns. However, up to 50% of treated newborns do not benefit from TH, particularly in low- and middle-income countries with perinatal infections counteracting HIE [3].

White matter injury is a common consequence of HIE and can lead to life-long neurological deficits such as cerebral palsy [4,5,6]. Such perinatal insults negatively impact oligodendrocyte maturation and cause myelination failure [7,8]. Thus, therapies to promote or accelerate myelination and oligodendrocyte proliferation could potentially ameliorate long-lasting neurological impairments after HIE [9]. Increased interest has emerged regarding the functional restoration of these cells as a potential therapeutic strategy for other diseases [10,11]; however, there is no clinical treatment available to prevent or cure WMI after HIE. A promising approach in the search for new neuroprotective treatments requires deeper understanding of the key molecular signals that affect oligodendrogenesis and myelination. The mitogen-activated protein kinase (MAPK) pathway involving extracellular signal-regulated kinase (ERK), p38, and Jun N-terminal kinase (JNK) is mainly involved in the regulation of inflammation, cellular stress, cell differentiation, and apoptosis [12]. From the three members of the MAPK pathway, the ERK cascade and its downstream transcriptional factors, such as the signal transducer and activator of transcription 3 (STAT3) and Interleukine-1 beta (IL-1 beta), play a major role in modulating oligodendrogenesis, particularly after brain damage [7,13,14].

Clemastine is a histamine H1 antagonist with anticholinergic properties against the muscarinic M1 receptor [15]. It has been demonstrated in pre-clinical studies that clemastine penetrates the blood–brain barrier and promotes re-myelination in animal models of white matter injury, multiple sclerosis, ischemia and Alzheimer’s disease [16,17,18,19]. Clemastine use in human was demonstrated in an adult case of delayed post-hypoxic leukoencephalopathy (DPHL) treated for 10 months, showing improvement on the white matter injury [20]. In neonates, few studies showed the effects of clemastine where the mothers were treated with anti-histaminic clemastine during breastfeeding, demonstrating irritability, but not neurological effects [21]. However, the use of clemastine in neonates is not well studied. Recently, we demonstrated that clemastine is a potential candidate for preventing HI brain injury in a neonatal rat model of HIE [22]. In the current study, we investigated the acute and long-term effects of clemastine in a neonatal rat model of hypoxic-ischemic injury and described the MAPK/ERK pathway as a potential mechanism underlying the neuroprotective effects of clemastine.

## 2. Results

### 2.1. Sex Specific Effects of Clemastine Treatment

We did not observe any sex differences between the treatment groups regarding area loss and the ERK pathway analyses (Supplementary Figure S1a–c,f). In addition, the long-term outcomes were not significantly different between the sexes. We observed a significant difference between the sexes in the analysis of oligodendrocytes (Olig2+ and CC1+ cells) after clemastine treatment in the hippocampal area 7 days after HI (Supplementary Figure S1d,e).

### 2.2. Clemastine Preserves Brain Tissue after Short- and Long-Term Survival

**Figure 1:**
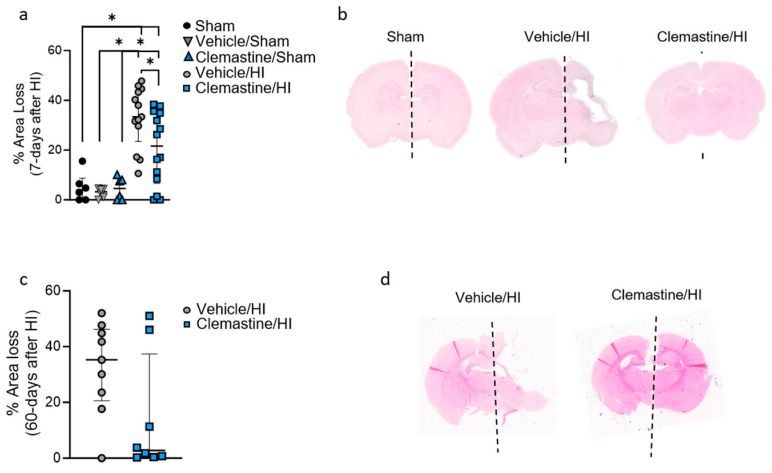
Neuroprotective effects of clemastine after hypoxia-ischemia. (**a**) Percentage of area loss 7 days after HI or sham animals treated with either vehicle or clemastine (10 mg/kg), single dose every 24 h for 6 consecutive days after HI. Sham n = 6 (black dots), vehicle/sham n = 6 (gray triangle), clemastine/sham n = 6 (blue triangle), vehicle/HI n = 13 (gray dots), and clemastine/HI n = 14 (blue squares). Sham vs. vehicle/HI * *p* = 0.0001, Mann–Whitney U = 1. Sham vs. clemastine/HI * *p* = 0.0307, Mann–Whitney U = 16. Vehicle/sham vs. Vehicle/HI * *p* < 0.0001, Mann–Whitney U = 0. Clemastine/sham vs. clemastine/HI *p* = 0.0241, Mann–Whitney U = 15. Vehicle/HI vs. clemastine/HI *p* = 0.0250, Mann–Whitney U = 45. (**b**) Hematoxylin and eosin staining, representing brain coronal section from sham (**left**) vs. vehicle/HI (**middle**) vs. clemastine/HI (**right**) 7 days after HI. (**c**) Percentage of area loss 60 days after HI in samples treated with either vehicle or clemastine (10 mg/kg), single dose every 24 h for 6 consecutive days after HI. Vehicle/HI n = 9 (gray dots) and clemastine/HI n = 8 (blue squares). (**d**) Hematoxylin and eosin staining, representing brain coronal section from vehicle/HI (**left**) vs. clemastine/HI (**right**) 60 days after HI. Nonparametric tests were performed using the Mann–Whitney U test with a 95% confidence interval. Data are expressed as the median (IQR).

**Figure 2:**
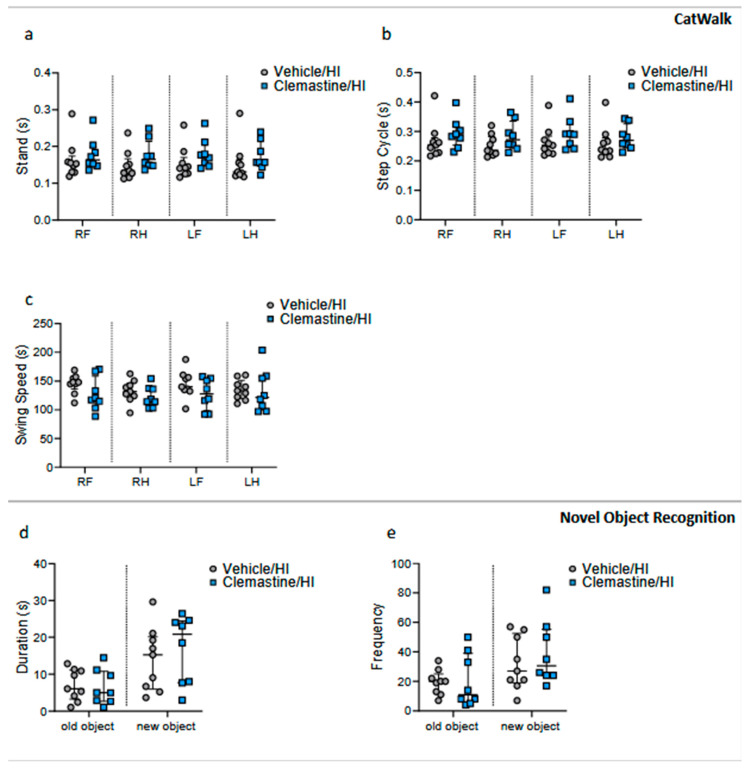
Motor and cognitive long-term effects of clemastine treatment. Neurobehavioral long-term results of the Catwalk test represented in (**a**) stand (s), (**b**) step cycle (s), and (**c**) swing speed (s) between the clemastine/HI and vehicle/HI groups. Neurobehavioral long-term results of the novel object recognition represented in (**d**) duration each animal spent at either the old or the new object, and (**e**) frequency at which the animals approached either the old or the new object, between the clemastine/HI and vehicle/HI groups. Vehicle/HI n = 9 (gray dots) and clemastine/HI n = 8 (blue squares). Nonparametric tests were performed using the Mann–Whitney U test with a 95% confidence interval. Data are expressed as the median (IQR).

We investigated the neuroprotective effects of clemastine by measuring the percentage of tissue loss in the affected brain hemisphere at two different time points after hypoxia ischemia. Seven days after HI, a significant increase in the percentage of area loss was observed when we compared the sham (3.9%), vehicle/sham (3.21%) and clemastine/sham (4.57%) groups to the vehicle/HI (33.14%) and clemastine/HI groups (20.54%) (Figure 1a,b; * *p* < 0.05. Sham: black dots, vehicle/sham: gray triangle, clemastine/sham: blue triangle, vehicle/HI: gray dots, and clemastine/HI: blue squares). Most important, at the same time point, a significant reduction in brain area loss was observed in the clemastine/HI group compared to that in the vehicle/HI group (20.54% vs. 33.14%, respectively) (Figure 1a,b; * *p* < 0.05. Vehicle/HI: gray dots and clemastine/HI: blue squares). No significant differences were observed in the analysis of percentage area loss in the cortex and hippocampal area when we compared the clemastine/HI group to that in the vehicle/HI group (Supplementary Figure S2. Vehicle/HI: gray dots and clemastine/HI: blue squares). However, a significant difference was observed when we compared the cortical and hippocampal area of the sham groups. The cortical sham (3.1%), sham/vehicle (3.79%), and clemastine/sham (6.74) groups showed a significant decrease in area loss compared to the cortical vehicle/HI and clemastine/HI groups (Supplementary Figure S2; * *p* < 0.05. Sham: black dots, vehicle/sham: gray triangle, clemastine/sham: blue triangle, vehicle/HI: gray dots, and clemastine/HI: blue squares). The same observation was found for hippocampal sham (11.22%), vehicle/sham (8.23%) and clemastine/sham (5.37%) groups compared to the hippocampal vehicle/HI and clemastine/HI groups (Supplementary Figure S2; * *p* < 0.05. Sham: black dots, vehicle/sham: gray triangle, clemastine/sham: blue triangle, vehicle/HI: gray dots, and clemastine/HI: blue squares). No significant tissue loss reduction was observed in the clemastine/HI group compared to the vehicle/HI group 60 days after HI (14.86% vs. 32%, respectively; Figure 2c,d. Vehicle/HI: gray dots, and clemastine/HI: blue squares).

### 2.3. Clemastine Effects on Long-Term Functional Outcome

We assessed the long-term motor and cognitive behaviors to determine the neuroprotective effects of clemastine. For this purpose, we preselected the animals using MRI 7 days after HI treatment with either the vehicle or clemastine. The majority of the damage observed was from the Bregma region 0.2 mm to Bregma −11.8 mm. As explained in the Materials and Methods Section, the score was based on the edema size and affected area. Of the 26 animals scanned, we kept n = 9 of the vehicle/HI group with a score of 3 and n = 8 of the clemastine/HI group with a score of 2.1 (Supplementary Figure S3a,b and Supplementary Table S1) for long-term behavior testing.

We performed the Catwalk test to assess motor behavior. For measurements related to the quality of the animals, such as stand, swing speed and step cycle, we did not see a significant difference between the clemastine/HI group and the vehicle/HI group (Figure 2a–c. Vehicle/HI: gray dots and clemastine/HI: blue squares). To assess cognitive improvement after treatment, we performed a novel object recognition test. We did not observe significant changes between the clemastine/HI group and the Vehicle/HI group (Figure 2d,e. Vehicle/HI: gray dots and clemastine/HI: blue squares).

### 2.4. Clemastine Neuroprotective Effects on Glia Cell Responses

**Figure 3:**
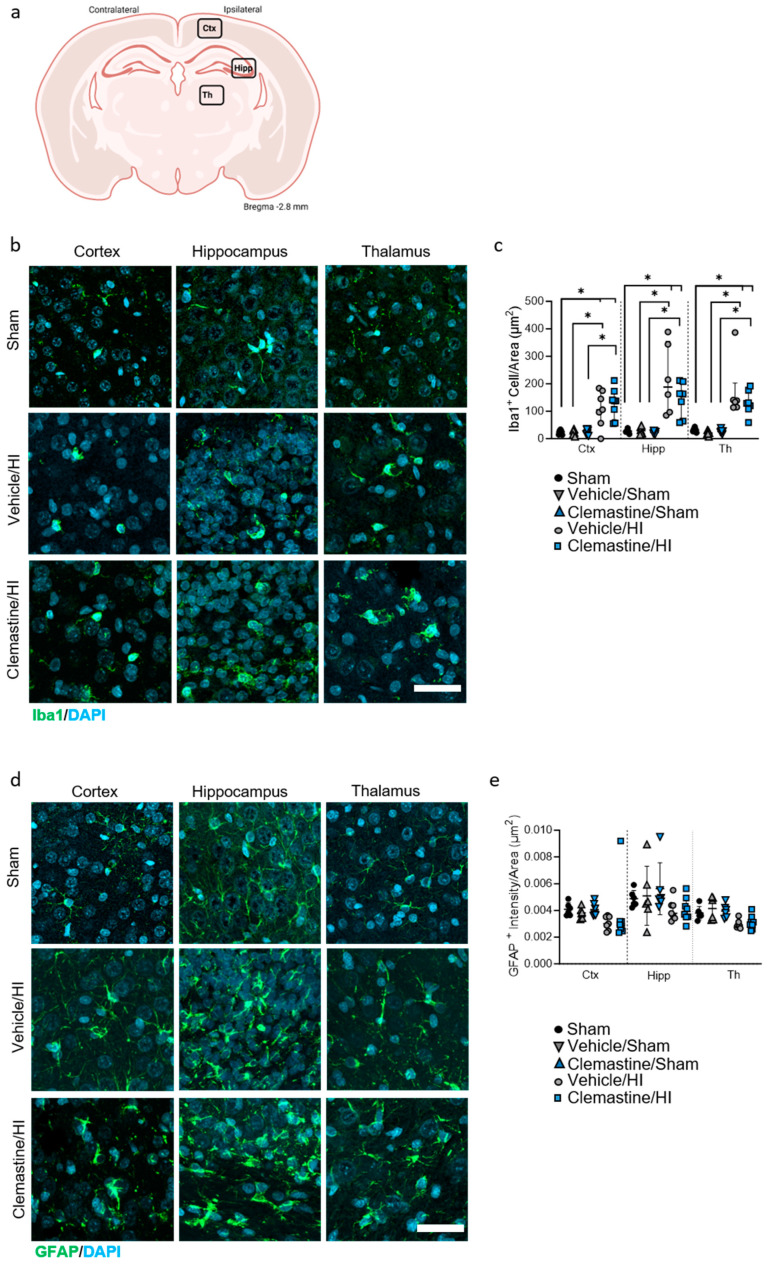
Clemastine effects on gliosis seven days after hypoxia ischemia. (**a**) Schematic image of the areas in the cortex, hippocampus, and thalamus used for quantitative analysis. (**b**) Representative images of the cortical (**left**), hippocampal (**middle**), and thalamic (**right**) areas stained for the microglia marker Iba-1 in green and the nuclear marker DAPI in blue. (**c**) Quantification of Iba-1+ cells in the cortex, hippocampus, and thalamus of the sham, vehicle/sham, clemastine/sham, clemastine/HI, and vehicle/HI groups. Sham n = 6 (black dots), vehicle/sham n = 6 (gray triangle), clemastine/sham n = 6 (blue triangle), vehicle/HI n = 7 (gray dots), and clemastine/HI n = 8 (blue squares). Cortex: sham vs. vehicle/sham * *p* = 0.0350 Mann–Whitney U = 6, sham vs. clemastine/HI * *p* = 0.0007 Mann–Whitney U = 0, vehicle/sham vs. vehicle/HI * *p* = 0.0007 Mann–Whitney U = 0, and clemastine/sham vs. clemastine/HI * *p* = 0.0007 Mann–Whitney U = 0. Hippocampus: sham vs. vehicle/sham * *p* = 0.0022 Mann–Whitney U = 0, sham vs. clemastine/HI * *p* = 0.0012 Mann–Whitney U = 0, vehicle/sham vs. vehicle/HI * *p* = 0.0022 Mann–Whitney U = 0, and clemastine/sham vs. clemastine/HI * *p* = 0.0012 Mann–Whitney U = 0. Thalamus: sham vs. vehicle/sham * *p* = 0.0022 Mann–Whitney U = 0, sham vs. clemastine/HI * *p* = 0.0012 Mann–Whitney U = 0, vehicle/sham vs. Vehicle/HI * *p* = 0.0022 Mann–Whitney U = 0, and clemastine/sham vs. clemastine/HI * *p* = 0.0012 Mann–Whitney U = 0. (**d**) Representative pictures of cortical (**left**), hippocampal (**middle**), and thalamic (**right**) area staining for the astrocyte marker GFAP in green and the nuclear marker DAPI in blue. (**e**) Quantification of GFAP+ cells in the cortex, hippocampus, and thalamus of the clemastine/HI and vehicle/HI groups. Sham n = 6 (black dots), vehicle/sham n = 6 (gray triangle), clemastine/sham n = 6 (blue triangle), vehicle/HI n = 7 (gray dots), and clemastine/HI n = 8 (blue squares). Nonparametric tests were performed using the Mann–Whitney U test with a 95% confidence interval. Data are expressed as the median (IQR). Scale bar 20 µm. Image (**a**) made with Biorender.com.

We further characterized the neuroprotective effects of clemastine on glia cell responses after HI using specific antibodies against microglia ionized calcium-binding adapter molecule 1 (Iba-1) and astrocytes glial fibrillary acidic proteins (GFAP). Analysis of Iba1+ in the cortex, hippocampus and thalamus showed a significant increase in the number of positive Iba-1 in the vehicle/HI and clemastine/HI groups compared to the sham, vehicle/sham, and clemastine/HI groups 7 days after HI (Figure 3a–c; * *p* < 0.05. Sham: black dots, vehicle/sham: gray triangle, clemastine/sham: blue triangle, vehicle/HI: gray dots, and clemastine/HI: blue squares). However, no significant changes in the number of positive Iba-1 cells were observed between the clemastine/HI and vehicle/HI groups (Figure 3a–c; * *p* < 0.05. Vehicle/HI: gray dots and clemastine/HI: blue squares). Analysis of astrogliosis did not show any significant differences between the areas and groups analyzed 7 days after HI (Figure 3d,e. Sham: black dots, vehicle/sham: gray triangle, clemastine/sham: blue triangle, vehicle/HI: gray dots, and clemastine/HI: blue squares).

### 2.5. Clemastine Neuroprotective Effect on Myelination

**Figure 4:**
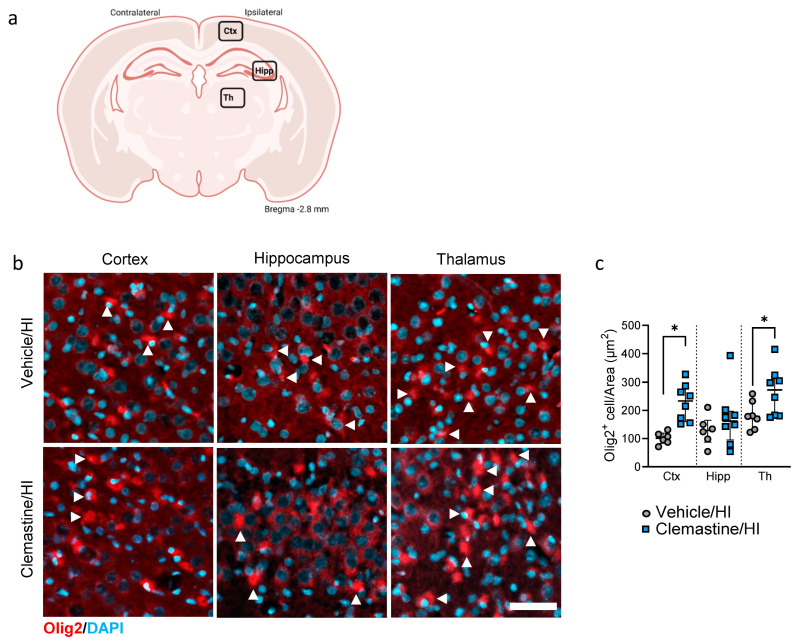

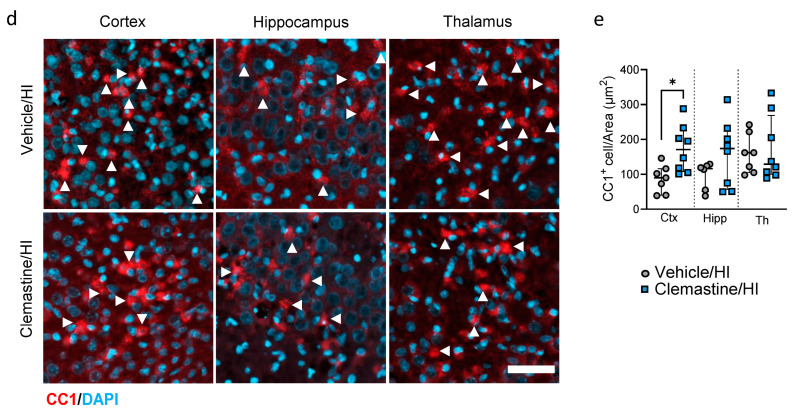
Clemastine effects on oligodendrocytes seven days after hypoxia ischemia. (**a**) Schematic image of the areas in the cortex, hippocampus, and thalamus used for quantitative analysis. (**b**) Representative images of the cortical (**left**), hippocampal (**middle**), and thalamic (**right**) areas stained for the immature oligodendrocyte marker Olig2 in red and nuclear marker DAPI in blue. (**c**) Quantification of Olig2+ cells in the cortex, hippocampus, and thalamus for the clemastine/HI and vehicle/HI groups. Vehicle/HI n = 7 (gray dots), and clemastine/HI n = 8 (blue squares). Cortex: vehicle/HI vs. clemastine/HI * *p* = 0.0007 Mann–Whitney U = 0. Thalamus: vehicle/HI vs. clemastine/HI * *p* = 0.0314 Mann–Whitney U = 9.5. (**d**) Representative images of the cortical (**left**), hippocampal (**middle**), and thalamic (**right**) areas stained for mature oligodendrocyte marker CC1 in red and nuclear marker DAPI in blue. (**e**) Quantification of CC1+ cells in the cortex, hippocampus, and thalamus for the clemastine/HI and vehicle/HI groups. Vehicle/HI n = 7 (gray dots) and clemastine/HI n = 8 (blue squares). Cortex: vehicle/HI vs. clemastine/HI * *p* = 0.0093 Mann–Whitney U = 6. Nonparametric tests were performed using the Mann–Whitney U test with a 95% confidence interval with a * *p* < 0.05. Data are expressed as the median (IQR). Scale bar 20 µm. Image (**a**) made with Biorender.com.

**Figure 5:**
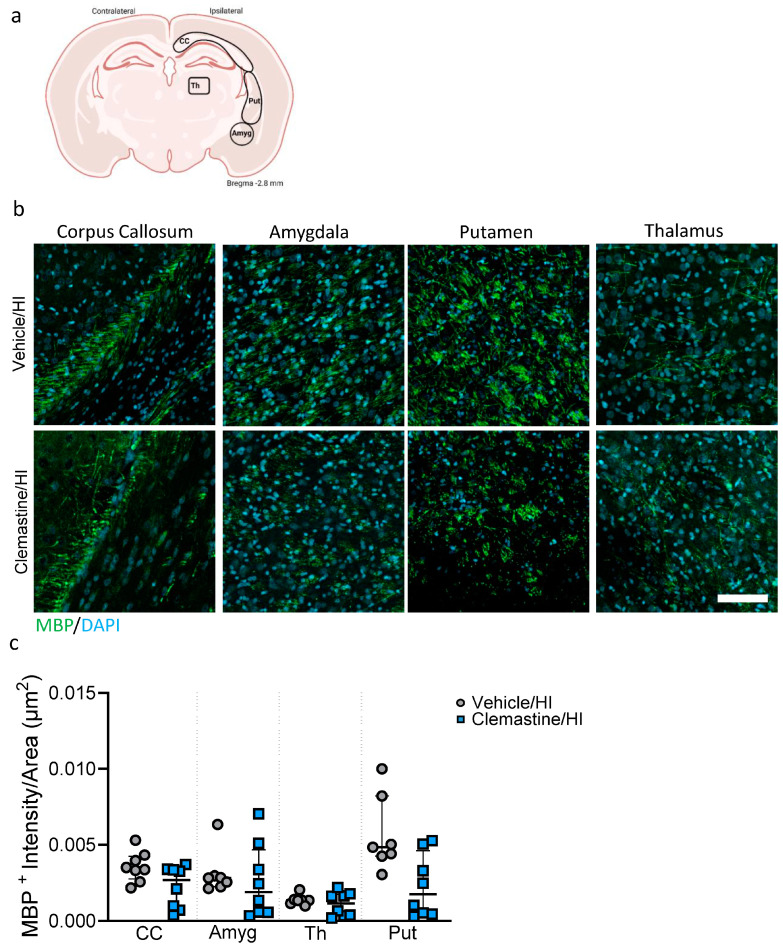
Clemastine effects on myelination seven days after hypoxia ischemia. (**a**) Schematic image of the areas in the corpus callosum, thalamus, putamen and amygdala used for quantitative analysis. (**b**) Representative images of the corpus callosum, amygdala, putamen, and thalamus for the staining of myelin basic proteins in green and the nuclear marker DAPI in blue. (**c**) The measured intensity by area of positive MBP signals in the corpus callosum, amygdala, putamen, and thalamus for the clemastine/HI and vehicle/HI groups. Vehicle/HI n = 7 (gray dots) and clemastine/HI n = 8 (blue squares). Nonparametric tests were performed using the Mann–Whitney U test with a 95% confidence interval. Data are expressed as the median (IQR). Scale bar 20 µm. Image (**a**) made with Biorender.com.

In this study, we characterized the effects of clemastine on myelination seven days after HI in the cortex, hippocampus and thalamus (Figure 4a). We observed a significant increase in the number of positive oligodendrocyte marker 2 (Olig2) in the cortex and thalamus of the clemastine/HI group compared to that in the vehicle/HI group (Figure 4b,c; * *p* < 0.05. Vehicle/HI: gray dots and clemastine/HI: blue squares). We analyzed mature oligodendrocytes using the oligodendrocyte marker anti-adenomatous polyposis coli (APC) clone CC1 (CC1). We observed a significant increase in mature CC1+ oligodendrocytes in the clemastine/HI group compared to the vehicle/HI group in the cortical area (Figure 4d,e, * *p* < 0.05. Vehicle/HI: gray dots and clemastine/HI: blue squares). We did not observe significant differences between the clemastine/HI and the vehicle/HI groups in the hippocampus and thalamus areas (Figure 4d,e. Vehicle/HI: gray dots and clemastine/HI: blue squares). We further analyzed the effect of clemastine on oligodendrocyte proliferation and survival using PathoGreen staining as a marker of cell death. We observed that the clemastine/HI group showed a significant decrease in the number of Olig2+/PathoGreen+ cells (labeled with arrowheads) in the cortex, hippocampus, and thalamus compared with the vehicle/HI group (Supplementary Figure S4a–c, * *p* < 0.05. Vehicle/HI: gray dots and clemastine/HI: blue squares). We also analyzed the effect of clemastine treatment on myelination by using the specific marker myelin basic protein (MBP) in the corpus callosum, thalamus, putamen and amygdala (Figure 5a). No significant differences were observed between the clemastine/HI and vehicle/HI groups in the areas analyzed (the corpus callosum, cortex, thalamus, and amygdala) (Figure 5b,c. Vehicle/HI: gray dots and clemastine/HI: blue squares).

### 2.6. Clemastine Neuroprotective Effect Is Mediated through the MAPK Pathway

**Figure 6:**
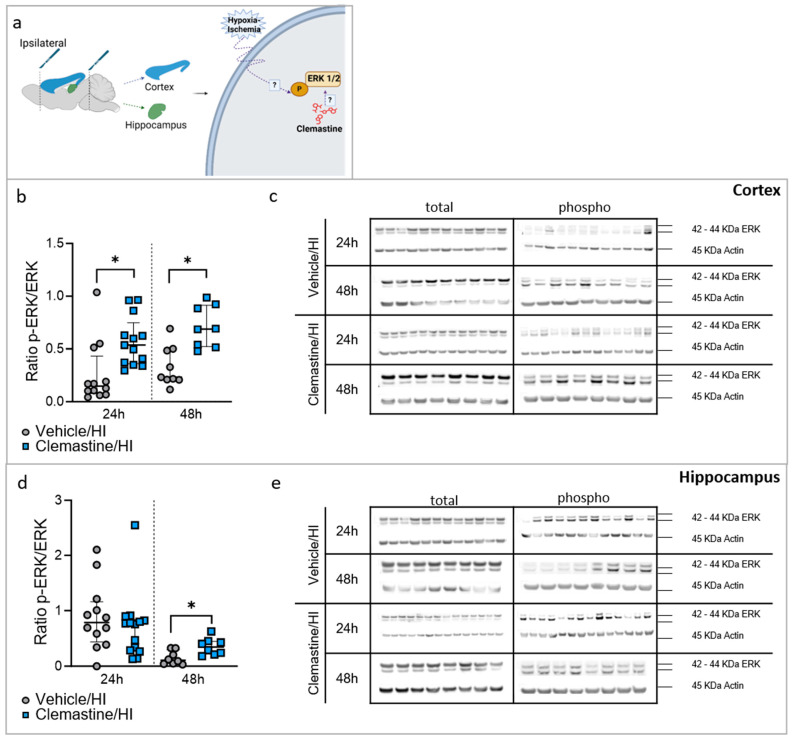
Neuroprotective effects of clemastine are mediated through the ERK1/2 pathway after HI. (**a**) Schematic image showing the dissection of the ipsilateral cortex and hippocampus used for biochemistry analysis. (**b**) Protein expression shown as the ratio of phosphorylated ERK (Thr202/Tyr204) compared to total ERK in the cortex. Cortex 24 h: vehicle/HI n = 12 (gray dots) and clemastine/HI n = 13 (blue squares). Cortex 48 h: vehicle/HI n = 9 (gray dots) and clemastine/HI n = 8 (blue squares). Time point: 24 h after HI: vehicle/HI vs. clemastine/HI * *p* = 0.0037 Mann–Whitney U = 26. 48 h after HI: vehicle/HI vs. clemastine/HI * *p* = 0.0026 Mann–Whitney U = 6.5. (**c**) Representative Western blot protein bands for ERK at 24 and 48 h in the cortex. Actin used as loading control. (**d**) Protein expression shown as the ratio of phosphorylated ERK (Thr202/Tyr204) compared to total ERK in the hippocampus. Hippocampus 24 h: vehicle/HI n = 12 (gray dots) and clemastine/HI n = 14 (blue squares). Hippocampus 48 h: vehicle/HI n = 8 (gray dots) and clemastine/HI n = 8 (blue squares). Time point: 48 h after HI: vehicle/HI vs. clemastine/HI * *p* = 0.0148 Mann–Whitney U = 9. (**e**) Representative protein bands of Western blot for ERK at 24 and 48 h in the hippocampus. Nonparametric tests were performed using the Mann–Whitney U test with a 95% confidence. Data are expressed as the median (IQR). Image (**a**) made with Biorender.com.

To elucidate the effects of clemastine on the MAPK pathway, we performed Western blotting of the ipsilateral side of the brain, particularly the cortex and hippocampus, 24 h and 48 h after HI (Figure 6a).

In the cortex, ERK expression (ratio of phosphorylated to total ERK) was significantly higher in the clemastine/HI group than in the vehicle/HI group at both time points (Figure 6b,c, * *p* < 0.05. Vehicle/HI: gray dots and clemastine/HI: blue squares). In the hippocampus, ERK expression showed a significant increase in the clemastine/HI group compared with the Vehicle/HI groups 48 h after HI (Figure 6d,e, * *p* < 0.05. Vehicle/HI: gray dots and clemastine/HI: blue squares).

Other members of the MAPK pathway, including the p38 and JNK, were also analyzed (Supplementary Figure S5a). The cortex did not show any significant differences between the clemastine/HI and vehicle/HI groups for either protein (Supplementary Figure S5b–e. Vehicle/HI: gray dots and clemastine/HI: blue squares). We observed a significant increase in p38 expression in the hippocampus in the clemastine/HI groups compared to the vehicle/HI groups 24 h after HI (Supplementary Figure S5f,g, * *p* < 0.05. Vehicle/HI: gray dots and clemastine/HI: blue squares). The analysis of JNK expression showed a significant decrease in the hippocampal area in the clemastine/HI group compared to the vehicle/HI group at both time points analyzed after HI (Supplementary Figure S5h,i, * *p* < 0.05. Vehicle/HI: gray dots and clemastine/HI: blue squares).

**Figure 7:**
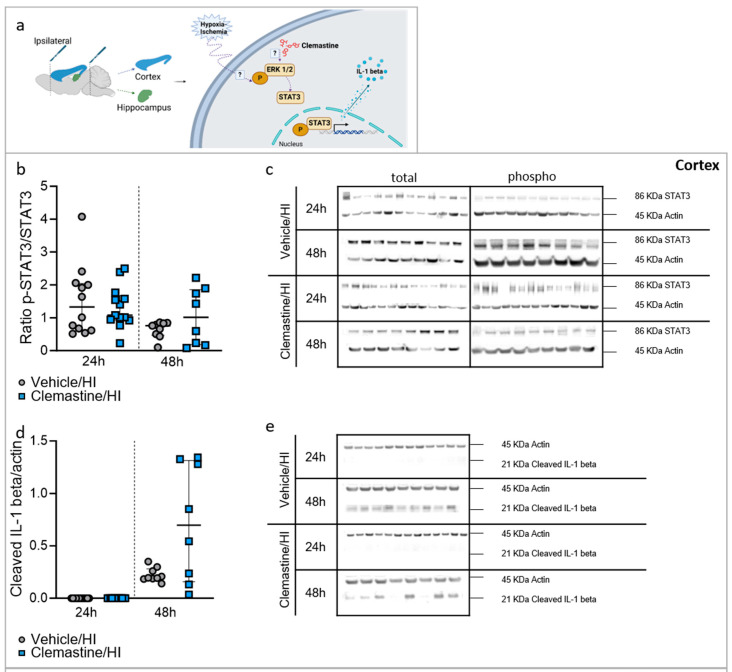

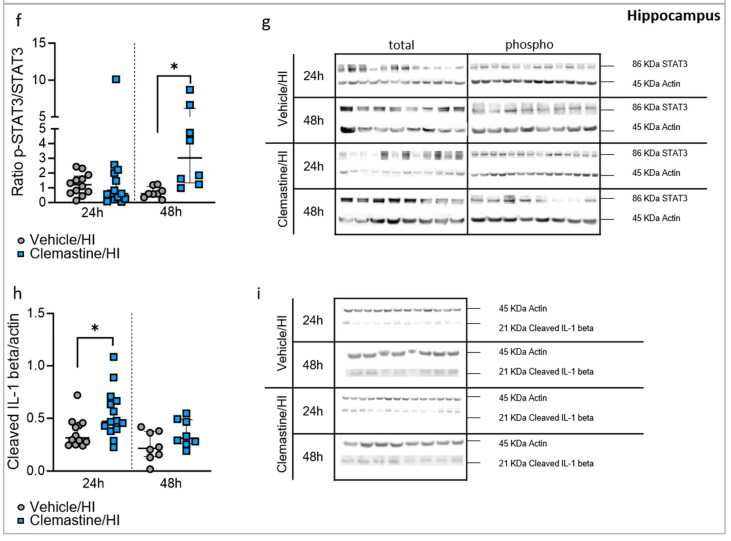
Neuroprotective effects of clemastine on downstream proteins from the ERK1/2 pathway after HI. (**a**) Schematic image showing the dissection of the ipsilateral cortex and hippocampus used for biochemistry analysis. (**b**) Protein expression was expressed as the ratio of phosphorylated STAT3 (Ser727) compared to total STAT3 in the cortex. Cortex 24 h: vehicle/HI n = 12 (gray dots) and clemastine/HI n = 13 (blue squares). Cortex 48 h: vehicle/HI n = 9 (gray dots) and clemastine/HI n = 8 (blue squares). (**c**) Representative Western blot protein bands for STAT3 at 24 and 48 h in the cortex. Actin used as loading control (**d**) Protein expression was expressed as the ratio of cleaved IL-1 beta normalized to actin as a loading control in the cortex. Cortex 24 h: vehicle/HI n = 12 (gray dots) and clemastine/HI n = 13 (blue squares). Cortex 48 h: vehicle/HI n = 9 (gray dots) and clemastine/HI n = 8 (blue squares). (**e**) Representative Western blot protein bands for cleaved IL-1 beta at 24 and 48 h in the cortex. Actin used as loading control. (**f**) Protein expression was expressed as the ratio of phosphorylated STAT3 (Ser727) compared to total STAT3 in the hippocampus. Hippocampus 24 h: vehicle/HI n = 12 (gray dots) and clemastine/HI n = 14 (blue squares). Hippocampus 48 h: vehicle/HI n = 8 (gray dots) and clemastine/HI n = 8 (blue squares). Time point: 48 h after HI: STAT3–vehicle/HI vs. clemastine/HI * *p* = 0.0006 Mann–Whitney U = 2. Time point: 24 h after HI IL-1beta–Vehicle/HI vs. clemastine/HI * *p* = 0.0407 Mann–Whitney U = 44. (**g**) Representative Western blot protein bands for STAT3 at 24 and 48 h in the hippocampus. Actin used as loading control (**h**) Protein expression was expressed as the ratio of cleaved IL-1 beta normalized to actin as a loading control in the hippocampus (*p* = 0.0407, Mann–Whitney U = 44 for 24 h TP). Hippocampus 24 h: vehicle/HI n = 12 (gray dots) and clemastine/HI n = 14 (blue squares). Hippocampus 48 h: vehicle/HI n = 8 (gray dots) and clemastine/HI n = 8 (blue squares). (**i**) Representative Western blot protein bands for cleaved IL-1 beta at 24 and 48 h in the hippocampus. Actin used as loading control. Nonparametric tests were performed using the Mann–Whitney U test with a 95% confidence interval. Data are expressed as the median (IQR). Image (**a**) made with Biorender.com.

We further analyzed the downstream components of the ERK pathway, such as STAT3 and IL-1 beta (Figure 7a). In the cortex, no significant difference in STAT3 between the clemastine/HI and vehicle/HI groups at either time point was observed (Figure 7b,c. Vehicle/HI: gray dots and clemastine/HI: blue squares). Additionally, IL-1 beta expression was higher (but not significant) in the clemastine/HI group than in the vehicle/HI group at 48 h after HI (Figure 7d,e; * *p* < 0.05. Vehicle/HI: gray dots and clemastine/HI: blue squares). In the hippocampus, STAT3 expression was significantly higher in the clemastine/HI group than in the vehicle/HI group 48 h after HI (Figure 7f,g; * *p* < 0.05. Vehicle/HI: gray dots and clemastine/HI: blue squares). Additionally, IL-1 beta expression was significantly higher in the clemastine/HI group than in the vehicle/HI group 24 h after HI (Figure 7h,i; * *p* < 0.05. Vehicle/HI: gray dots and clemastine/HI: blue squares).

## 3. Discussion

Our results showed a neuroprotective effect of clemastine on the injured brain after hypoxia-ischemia in a neonatal rat model of HIE. We found that clemastine preserved oligodendrocyte proliferation in our neonatal model of hypoxic-ischemic brain injury. We demonstrated that the neuroprotective effects observed after clemastine treatment involved regulation of the MAPK/ERK pathway, with regulation of downstream effectors, such as STAT3 and IL-1 beta.

White matter injury (WMI) is a common consequence after HIE and can lead to life-long neurological deficits such as cerebral palsy, and cognitive and persistent motor disabilities [6,23,24,25]. Although WMI is mainly associated with premature birth [9], it may also occur in term infants following HIE. White matter injury negatively affects the proliferation and maturation of oligodendrocytes and causes myelination failure [25]. Although treatment with therapeutic hypothermia is an effective approach for HIE [26], several studies have demonstrated that TH does not have a beneficial effect on WMI [27,28,29]. Currently, there is no clinical treatment available to prevent or cure WMI, making the discovery of new or alternative treatments important.

Clemastine is a first-generation histamine H1 receptor antagonist and it is approved for usage in children [15]. Although, initially, clemastine was primarily focused on its antihistaminic properties, several studies have recently identified it as a potential treatment for multiple sclerosis (MS) through promoting remyelination [30], as well as for other neurodevelopmental deficits [31,32,33,34]. In the context of hypoxia-related brain damage, clemastine showed to be neuroprotective in an adult model of bilateral common carotid artery occlusion [35] and in a preterm model of delayed post-hypoxic leukoencephalopathy [20]. However, we recently demonstrated the potential neuroprotective effects of clemastine in neonatal hypoxia-ischemia [22]. Nevertheless, a full appreciation of the therapeutic potential of clemastine in neonatal hypoxia-ischemia disorders is needed to reveal the underlying mechanisms.

Our results showed a significant reduction in white matter loss 7 days and 60 days after HI in animals treated with clemastine, confirming the long-term neuroprotective effect of clemastine in our model of HIE. Nevertheless, we did not observe any motor or cognitive improvement after clemastine treatment at the time of the analysis. Based on these data, a limitation of our study can be explained by the dose used (daily injection of 10 mg/kg for 6 consecutive days), which may explain the lack of improvement in motor and cognitive levels. Several studies indicate the need for several daily doses of clemastine (up to 14 days) to achieve maximum and long-lasting neuroprotective effects [31,32,36,37]. Recently, it was demonstrated in a preterm white matter injury mouse model that clemastine rescued hypoxia-induced hypomyelination at a single daily dose of 7.5 mg/kg for 7 consecutive days and pharmacokinetic analysis revealed a C_max_ of 44.0 ng/mL, t_1/2_ 4.6 h [18]. Based on this study, our dosage regimen was chosen (after personal communication with the authors). Thus, there is a need to perform further studies regarding the dose regimen and appropriate behavioral time to test the most efficacious neuroprotective effects of clemastine in our model.

Myelination is a continuous process that begins shortly before a term neonate is born, peaks after birth during the first years of life, and continues to refine into adolescence [38]. Therefore, any event leading to brain injury, such as hypoxia-ischemia, may impair this process and cause long-term disability [38]. Oligodendrocyte precursor cells are located in the subventricular zone, where they can migrate to their target location and differentiate into mature oligodendrocytes, playing a key role in the myelination process [39]. We observed a significant increase in the number of immature oligodendrocytes Olig2+ in the cortex and thalamus. A similar effect was observed with mature oligodendrocytes, CC1+ cells, and myelination at the time of analysis in the cortical areas and adjacent corpus callosum.

As oligodendrogenesis is a process that requires time to develop, particularly after a brain injury such as HIE, the time point analyzed in this study might not be the best to determine further functional oligodendrocyte and myelination maturation. Previous studies on oligodendrogenesis have been published in different models of brain injury treated with clemastine [20,33,37]; however, most studies have been performed in adult animal models. Therefore, further studies are needed to shed light on how clemastine improves myelination and long-term outcomes in neonates with HIE. In a mouse model of HIE, hypoxia-ischemia can have a long-term dynamic effect on the endogenous oligodendrogenesis of neonatal rat brain white matter, where the number of mature oligodendrocytes recovered to the normal level 56 to 84 days after HI, but myelination was still blocked, which suggests that it is essential to promote the maturation of oligodendrocytes and their functional recovery [40]. Similar results were observed in human neonates undergoing HIE with a dramatic decrease in myelination in the posterior limbs of the internal capsule, thalami, and lentiform nuclei, as well as optic radiations, and in the anterior/posterior white matter [41].

An important feature of HIE is an increase in gliosis, which predicts the outcome of neonates undergoing HIE [42]. We did not observe significant differences in microgliosis or astrogliosis after clemastine treatment, suggesting that clemastine did not directly affect the resolution of inflammation after HIE. However, there is little evidence that clemastine is a potential regulator of microglial and astrocyte [43] priming. However, it has been shown in primary cells [35,37], highlighting the need for further studies to understand the exact role of clemastine in inflammation, particularly because glial cells play a key role in oligodendrogenesis [44,45].

During normal homeostasis and stress stimuli, such as hypoxia-ischemia, many molecular pathways are involved not only in the resolution of inflammation but also in the survival and regeneration of the affected cells [46,47,48]. One of these pathways is the MAPK pathway, which includes the downstream targets ERK, p38, and JNK. Furthermore, the ERK signaling pathway plays a central role in the cellular program of myelination, including the proliferation of oligodendrocytes to enhance myelin growth [13]. However, the exact molecular pathways involved in oligodendrogenesis and myelination in neonatal hypoxia-ischemia remain unclear. In the present study, we observed significant changes in the expression level of the ERK pathway, which was more pronounced 48 h after hypoxia in both areas analyzed. Interestingly, downstream effectors were significantly regulated, particularly in the hippocampal area. IL-1beta is secreted by oligodendrocytes and regulates their proliferation and differentiation [49]. It has been demonstrated that, unlike other pro-inflammatory cytokines, IL-1beta is not toxic for oligodendrocyte linage cells [49]. However, it is not clear if after an injury such as hypoxia-ischemia, the levels of IL-1beta could inhibit proliferation of oligodendrocytes progenitors and later myelination as it was previously demonstrated in a model of periventricular white matter damage [43]. In this context, excess IL-1beta was demonstrated to inhibit the maturation of progenitor oligodendrocytes via the ERK pathway thereby leading to hypomyelination [43]. It is important to note that the analysis performed in this study is from brain homogenates and not isolated oligodendrocyte cells, highlighting the importance of future experiments to elucidate the exact role of the MAPK pathway and IL-1beta in individual cell types by using specific inhibitors.

In a model of spinal cord injury, clemastine was shown to promote oligodendrogenesis through activation of the ERK pathway [50]. In the same study, it was shown that inhibition of ERK in a spinal cord injury mouse model treated with clemastine decreased the number of mature oligodendrocytes, suggesting that clemastine effects on oligodendrogenesis require the ERK pathway [50].

## 4. Material and Methods

### 4.1. Animal and Experimental Procedures

All experiments were performed in accordance with ARRIVE guidelines and under the supervision of the Animal Protection Committee of the North Rhine-Westphalia State Environment Agency (LANUV), Germany. The animal’s exposure was regulated with a 12:12 dark/light cycle at room temperature (21 °C), and food and water were provided ad libitum. The premises and organization were provided by the Central Animal Laboratory of the Deutsches Zentrum für Neurodegenerative Erkrankungen (DZNE) Bonn, Germany. Seven-day-old Wistar rat pups of both sexes were used in this study. Fifteen female Wistar rats were used with an average litter size of 10–12 pups. Animals were randomized across litters, sexes, and weights for all the treatments. Those who performed the experiments and analyses were blinded to the specific treatments. A total of 141 animals were used in five different treatment groups and sacrificed at two different time points: P14 and P60 (Table 1). To maintain a standardized procedure, the established Vannucci model was used in all experiments [51,52]. Under general isoflurane anesthesia, the left common carotid artery was ligated, followed by 90 min of hypoxia (8% O_2_) at a rectal temperature (Trectal) of 36 °C. This resulted in a moderate hypoxic-ischemic (HI) brain injury. After the procedure, all animals were exposed to normothermia (NT) treatment, maintaining a Trectal of 37.0 °C for 5 h. Following the treatment period, the pups were returned to their dams and sacrificed at different time points (Supplementary Figure S6). During the experiments, temperature was monitored using a rectal probe (IT-21, Physitemp Instruments, Clifton, NJ, USA) connected to a servo-controlled cooling machine and a cooling mat (CritiCool, MTRE, Yavne, Israel). Temperatures during the experiments were controlled by a “sentinel” rat pup that was not in the treatment groups as previously described ([51,52]. Sham animals were treated only with 5 min of Isoflurane. The animals were treated daily with a single intraperitoneal injection (i.p.) of clemastine (10 mg/kg [22]) or vehicle (NaCl 0.9%) for 6 consecutive days (Supplementary Figure S6), where the first dose was immediately after HI.

**Area Loss analysis.**

At seven or sixty days post HI (P14 (3.TP) and P60 (4.TP)), n = 51 for P14 and n = 28 for P60 (for each condition, see Table 1 for exclusion criteria), animals were euthanized and transcardiac perfusion with 10% neutral formalin (Sigma, Germany) was performed as previously described [22]. The brains were then further processed into coronal blocks, cut into 3 mm sections, and embedded in paraffin. These sections were cut into 10 µm-thick slices, taken from two adjacent blocks representing different brain regions, including the cortex, hippocampus, basal ganglia, and thalamus, measured at a distance of −3.8 mm from the bregma. Hematoxylin and eosin (HE) staining were performed to determine the white matter loss, and the resulting slices were digitally scanned using an Epson Perfection V750 Pro scanner. The ImageJ software was used to analyze the optical density and area of the hemisphere. This analysis involved comparing the ipsilateral side with the contralateral side, and the extent of area loss on the ligated side was calculated using the formula (1 − (area left/area right) × 100), as established in prior research studies [22,52]. This methodology for the analysis of area loss was previously validated by a neuropathologist which scored the animals based in a validated scored system of a 9-step scale from 0.0 (no injury) to 4.0 (>90% injury). Based on this, a pathology score of ~50% correlates to our computer-calculated area loss of ~40%. Based on this, a 60% area loss corresponds to a ~3.5–4 pathology score [53]

### 4.2. Magnetic Resonance Image (MRI)

To assess the extent of damage seven days after hypoxia ischemia and to pre-select the animals used for long-term behavior, MRI scans were performed and analyzed [54]. We used an MRI with a 11.7 Tesla (T) horizontal small-bore magnet (Biospec 117/16, Bruker, Billerica, MA, USA) equipped with a rat brain receive-only proton (1H) coil from Bruker Biospin. The images were taken using a rapid acquisition relaxation enhancement (RARE) T2-weighted (T2-w) sequence with the following parameters: echo time (TE) = 25 ms, repetition time (TR) = 2.9 s, and in-plane resolution of 0.156 × 0.156 mm^2^. A sagittal plane from anterior to posterior was used, with a primary focus on the Bregma region spanning from 0.2 mm to −11.8 mm. The animals were classified according to the following scale by analyzing the volume of the edema and the affected area: 1, no damage; 2, mild damage; 3, moderate damage; and 4, severe damage. A total of 28 animals were scanned, with an injury around the median (Vehicle/HI = 2.4 and clemastine/HI = 2.7). Nine animals were kept for behavioral testing in the vehicle group (median damage score 3) and eight in the clemastine treatment group (median damage score 2.1) (Table 1 and Table S1).

### 4.3. Long-Term Behavioral Testing

To examine the long-term effects of clemastine, two behavioral tests were conducted. Novel object recognition was used to assess cognitive function and the Catwalk test was used to assess motor function. In both cases, the animals underwent a two-day training period, with the actual test taking place on the third day. The Catwalk test was conducted between postnatal days 46–48 and the novel object recognition test was conducted between postnatal days 53–55.

- Catwalk: Each animal ran three tests, with a maximum of 5 s (to be considered successful). The camera captured the animal’s gait from below, and the glass plate was illuminated in red to ensure a more detailed step analysis. We closely examined the run speed (cm/s), step cycle (time (s) from when an initial paw contacts the glass to the next time the paw meets the glass), stand (duration (s) of contact of a paw with the glass plate), and swing speed (speed (distance unit/second) of the paw during swing) using Catwalk XT 10.6 software and its corresponding analysis system (Nodulus XT). These parameters were based on average measurements from all three runs.
- Novel object recognition (NOR): The test was performed in four 45 × 45 cm boxes with white walls and a black floor, as previously described [52]. On training days, each rat was placed in the same empty box for 5 min for habituation, with two identical objects placed at opposite corners. On day three, each rat was placed in the same box with two identical objects for 5 min. After a pause, each rat was placed in the same box, where one of the objects was replaced with a novel object for another 5 min. The test was recorded on video and analyzed using an EthoVision XT 17.5 analysis system (Noldus Information Technology, Wageningen, The Netherlands). For the analysis, only the data from the third interval were used. The analysis involved calculating the ratio of time the animal spent in the new object’s area to the old one. Additionally, the frequency with which the animal approached either the old or the new object was analyzed.

### 4.4. Immunohistochemistry (IHC)

Six sham, 6 Vehicle/Sham, 6 clemastine/Sham, 7 Vehicle/HI, and 8 clemastine/HI treated animals were subjected to immunohistochemically analysis 7 days after HI (Table 1). The tissues were treated as previously described [52]. The slides were treated with 0.1% Triton X-100 for 30 min at room temperature, followed by blocking with 20% normal goat serum (Invitrogen). The primary antibodies were diluted and incubated overnight at 4 °C (Table 2). Appropriate secondary antibodies (Table 2) were used depending on the primary antibody. Both primary and secondary antibodies were diluted in a 0.7% carrageenan solution. The sections were counterstained with 4,6-diamidino-2-phenylindole (DAPI) (Invitrogen). Visualization of the IHC was performed utilizing fluorescence microscopy using AxioScan Z.1 (Carl Zeiss Microscopy GmbH, Munich, Germany) with a 20× objective, as well as a confocal microscope LSM900 (Zeiss, Germany) with a 20× objective. The analysis was performed using ZEN Blue 3.1 (Carl Zeiss Microscopy GmbH, Munich, Germany) and ImageJ software (USA), focusing on the following areas: the frontoparietal cortex (motor cortex), hippocampus (CA2-CA3), and ventroposterior thalamus for the analysis of Iba1, GFAP, Olig2, CC1, and PathoGreen. The corpus callosum, amygdala, putamen, and thalamus were used for MBP analysis. For analysis, the ipsilateral side was scanned with a z-stack of 3 µm (intervals of 3 µm each). After scanning, background subtraction was performed using the Zen Blue software (Carl Zeiss Microscopy GmbH, Munich, Germany), followed by depth focus extension to stitch all z-slices into one. The number of positively responding cells was quantified using DAPI staining and the intensity of the responsive antibody in relation to the area was measured. Oligodendrocyte proliferation and cell death was quantified using PathoGreen and Olig2 immunostaining, respectively, and results were normalized to the area.

### 4.5. Biochemistry Analysis

At 24 h and 48 h after hypoxia (1.TP and 2.TP, respectively), the brain samples were dissected from the cortex and hippocampus, and immediately snap-frozen (Table 1). For analysis, different brain samples were lysed using a radioimmunoprecipitation (RIPA) buffer (Millipore) with the addition of phosphatase and protease inhibitors (Roche). The exact protein concentration was determined using a bicinchoninic acid assay (BCA Pierce, Thermo Fisher Scientific, Germany). For Western blotting, 50 µg of each sample was loaded onto a 10% Bis-Tris gel. SDS-PAGE was performed in a 2-Morpholinoethanesulfonic acid (MES) buffer system (Thermo Fisher Scientific) and then transferred to a polyvinylidene difluoride membrane overnight at 4 °C at 30 V. The membranes were blocked with a 5% (*w/v*) non-fat milk solution (for non-phosphorylated proteins) or a 5% (*w/v*) bovine serum albumin (BSA) (for phosphorylated proteins) in TBS with 0.05% (*v/v*) Tween 20 (MP Biomedical, Germany) for 1 h at room temperature. The membranes were incubated with primary antibodies (Table 2) overnight at 4 °C. The blots were scanned using an Odyssey infrared imaging system (LI-COR Biosciences, Germany). The optical density of the bands was determined using the ImageJ software and normalized to the optical density of β-actin as a control. The ratio was calculated based on the amount of phosphorylated protein compared with the total amount of the corresponding protein.

### 4.6. Statistical Analysis

All numerical values are presented as the median and interquartile range (IQR). All analyses and data plots were generated using GraphPad Prism 6 (GraphPad Software, La Jolla, CA, USA). For the analysis of protein expression (MAPK pathway) at different time points after clemastine treatment and for immunohistochemistry analysis, nonparametric tests were performed using the Mann–Whitney test with a 95% confidence interval. For area loss and behavioral tests, nonparametric tests were performed using the Mann–Whitney U test with a 95% confidence interval. All animals were randomized according to litter, sex, and weight at the time of the experiment. Linear regression analysis was performed to determine the effects of sex and weight gain on the individual results. In all of our results, statistical significance was set at * *p* < 0.05 or *** *p* < 0.0001.

## 5. Conclusions

Our results provide new insights into the effect of clemastine on neonatal HIE and the mechanisms involved in this neuroprotective effect. We demonstrated that the ERK pathway, along with regulation of the transcriptional factor STAT3, was involved in the neuroprotective effects of clemastine. This study demonstrated that clemastine treatment following HIE enhanced oligodendrogenesis and reduced white matter injury. These results show that clemastine is a promising therapeutic agent for HIE, especially in LMIC, where TH is not readily available or does not show promising results.

## Figures and Tables

**Table 1:** Number of animals used per condition.

Purpose	Time Point (after HI)	Treatment (after HI)	N Animal Experiment	N Mortality	N Analysis	N Excluded Analysis	♂-♀ Included Analysis
Western Blot	24 h	Vehicle/HI	30	0	15	3 * Cortex and 3 * Hippocampus	Cortex (♂5-♀7)-Hippocampus (♂5-♀7)
		Clemastine/HI			15	2 * Cortex and 7 * Hippocampus	Cortex (♂7-♀6)-Hippocampus (♂3-♀5)
	48 h	Vehicle/HI	32	5	13	4 * Cortex and 4 * Hippocampus	Cortex (♂5-♀4)-Hippocampus (♂5-♀4)
		Clemastine/HI			14	5 * cortex and 5 * Hippocampus	Cortex (♂3-♀6)-Hippocampus (♂3-♀6)
Area loss/IHC	7 days	Sham	6	0	6	0	♂0-♀6
		Vehicle/Sham	6	0	6	0	♂2-♀4
		Clemastine/Sham	6	0	6	0	♂2-♀4
		Vehicle/HI	33	6	13	0	♂8-♀5
		Clemastine/HI			14	0	♂6-♀8
	46–55 days	Vehicle/HI	28	2	13	4 ** MRI	♂4-♀5
		Clemastine/HI			13	5 ** MRI	♂4-♀5
Behavior		Vehicle/HI	28 (same animals as area loss/IHC 60 days)	2	13	4 ** MRI	♂4-♀5
		Clemastine/HI			13	5 ** MRI	♂4-♀5
IHC ****	7 days	Sham	6	0	6	0	♂0-♀6
		Vehicle/Sham	6	0	6	0	♂2-♀4
		Clemastine/Sham	6	0	6	0	♂2-♀4
		Vehicle/HI	33 (same animals as area loss/IHC 7 days)	6	13	6 ***	♂3-♀4
		Clemastine/HI			14	6 ***	♂4-♀4

Mortality: death due to hypoxia; * excluded due not enough protein for biochemistry analysis or areas were damaged for dissection; ** excluded after MRI; *** too much damage for analysis; **** same animals as Area loss/ICH; ♂: Male; ♀: Female.

**Table 2:** Antibody list.

Technique	Cat. N°	Antibody	Supplier	Clone	Dilution WB	Dilution IHC	Host
Western Blot	9102	p44/42 MAPK (Erk1/2)	Cell Signaling	-	1 in 500	-	rabbit
	4511	phospho p44/42 MAPK (Erk 1/2) (Thr202/Tyr204)	Cell Signaling	D13.14.4E	1 in 500	-	rabbit
	9212	p38 MAPK	Cell Signaling	-	1 in 500	-	rabbit
	2855	phospho p38 MAPK (Thr180/Tyr182)	Cell Signaling	D3F9	1 in 500	-	rabbit
	9252	SAPK/JNK	Cell Signaling	-	1 in 500	-	rabbit
	4668	phospho SAPK/JNK (Thr183/Tyr185)	Cell Signaling	81E11	1 in 500	-	rabbit
	30835	Stat3	Cell Signaling	D1B2J	1 in 500	-	rabbit
	5364	phospho Stat3 (Ser727)	Cell Signaling	-	1 in 500	-	rabbit
	26048-1-AP	IL-1beta	Proteintech	-	1 in 200	-	rabbit
	A1978	β-actin	Sigma	AC-15	1 in 3000	-	mouse
	35518	goat-anti-mouse IgG (H+L) DyLight^TM^ 680 Conjugated	Invitrogen	-	1 in 3000	-	mouse
	SA535571	goat-anti-rabbit IgG (H+L) DyLight^TM^ 800 Conjugated	Invitrogen	-	1 in 3000	-	rabbit
IHC	019-19741	Iba-1	Wako	-	-	1 in 300	rabbit
	ABIN3183309	Anti-APC (CC1)	Antikorper-online.de	-	-	1 in 100	rabbit
	AB9610	Oli-2	Millipore	-	-	1 in 200	rabbit
	80788	GFAP	Cell Signaling	E4L7M	-	1 in 100	rabbit
	ab218011	Myelin Basic Protein (MBP)	Abcam	EPR21188	-	1 in 500	rabbit
	A11008	goat-anti-rabbit IgG (H+L) Cross-Adsorbed Alexa Fluor^TM^ 488	Invitrogen	-	-	1 in 500	rabbit

## Data Availability

All data are accessible via the corresponding author.

## Supplementary Materials

The following supporting information can be downloaded at: https://www.mdpi.com/article/10.3390/ijms25158204/s1.
