# Supplementary material for: Neuroprotective Effect of Clemastine Improved Oligodendrocyte Proliferation through the MAPK/ERK Pathway in a Neonatal Hypoxia Ischemia Rat Model
Source: Int J Mol Sci. 2024 Jul 27;25(15):8204. doi: 10.3390/ijms25158204 (PMC11311837; doi:10.3390/ijms25158204)
Supplement: Supplementary file 1 [file ijms-25-08204-s001.zip › Clemastine Supplementary Data.pptx]

## Slide 1
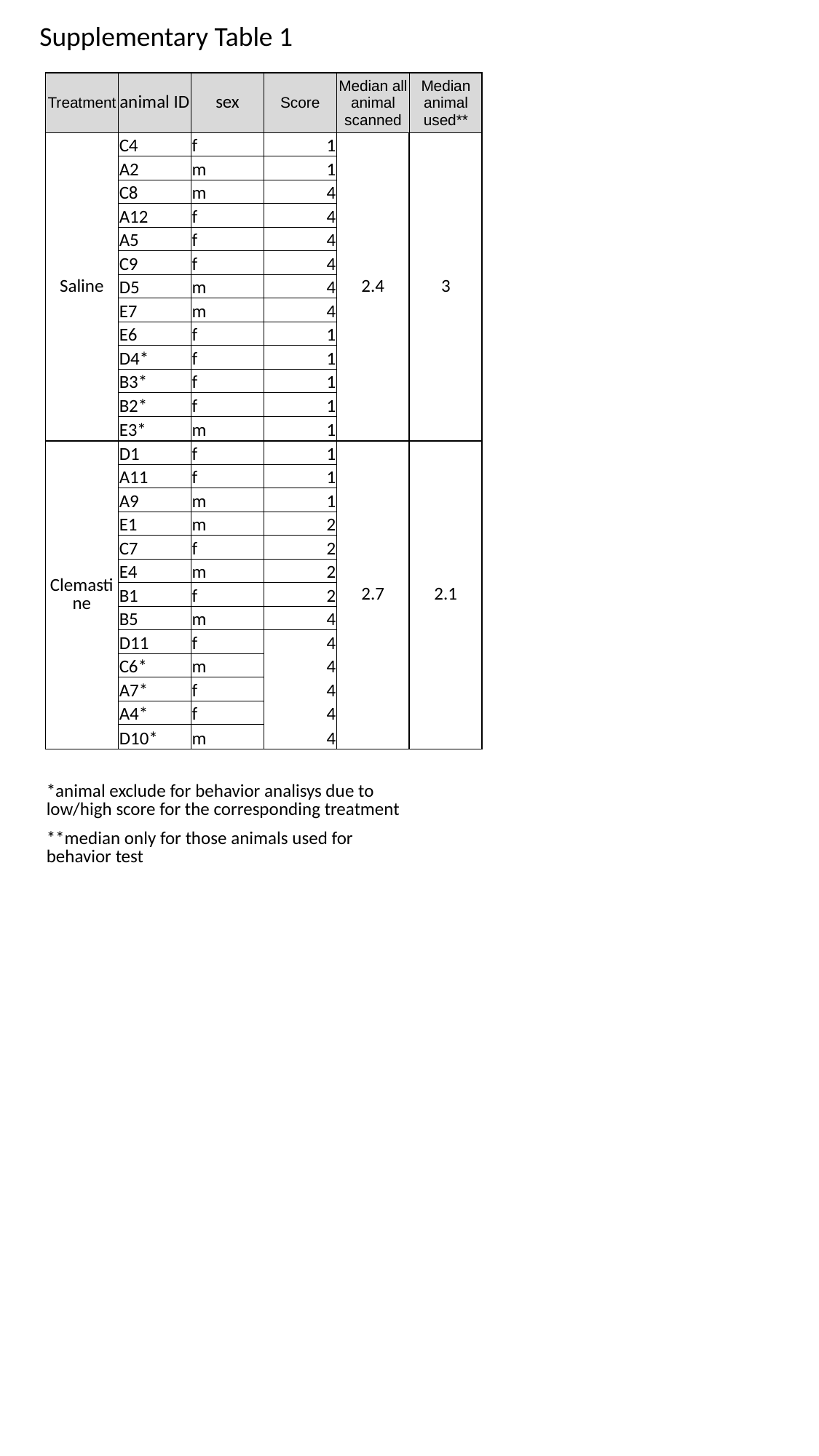

Supplementary Table 1
| Treatment | animal ID | sex | Score | Median all animal scanned | Median animal used\*\* |
| --- | --- | --- | --- | --- | --- |
| Saline | C4 | f | 1 | 2.4 | 3 |
| | A2 | m | 1 | | |
| | C8 | m | 4 | | |
| | A12 | f | 4 | | |
| | A5 | f | 4 | | |
| | C9 | f | 4 | | |
| | D5 | m | 4 | | |
| | E7 | m | 4 | | |
| | E6 | f | 1 | | |
| | D4\* | f | 1 | | |
| | B3\* | f | 1 | | |
| | B2\* | f | 1 | | |
| | E3\* | m | 1 | | |
| Clemastine | D1 | f | 1 | 2.7 | 2.1 |
| | A11 | f | 1 | | |
| | A9 | m | 1 | | |
| | E1 | m | 2 | | |
| | C7 | f | 2 | | |
| | E4 | m | 2 | | |
| | B1 | f | 2 | | |
| | B5 | m | 4 | | |
| | D11 | f | 4 | | |
| | C6\* | m | 4 | | |
| | A7\* | f | 4 | | |
| | A4\* | f | 4 | | |
| | D10\* | m | 4 | | |
| \*animal exclude for behavior analisys due to low/high score for the corresponding treatment | | | | | |
| \*\*median only for those animals used for behavior test | | | | | |

## Slide 2
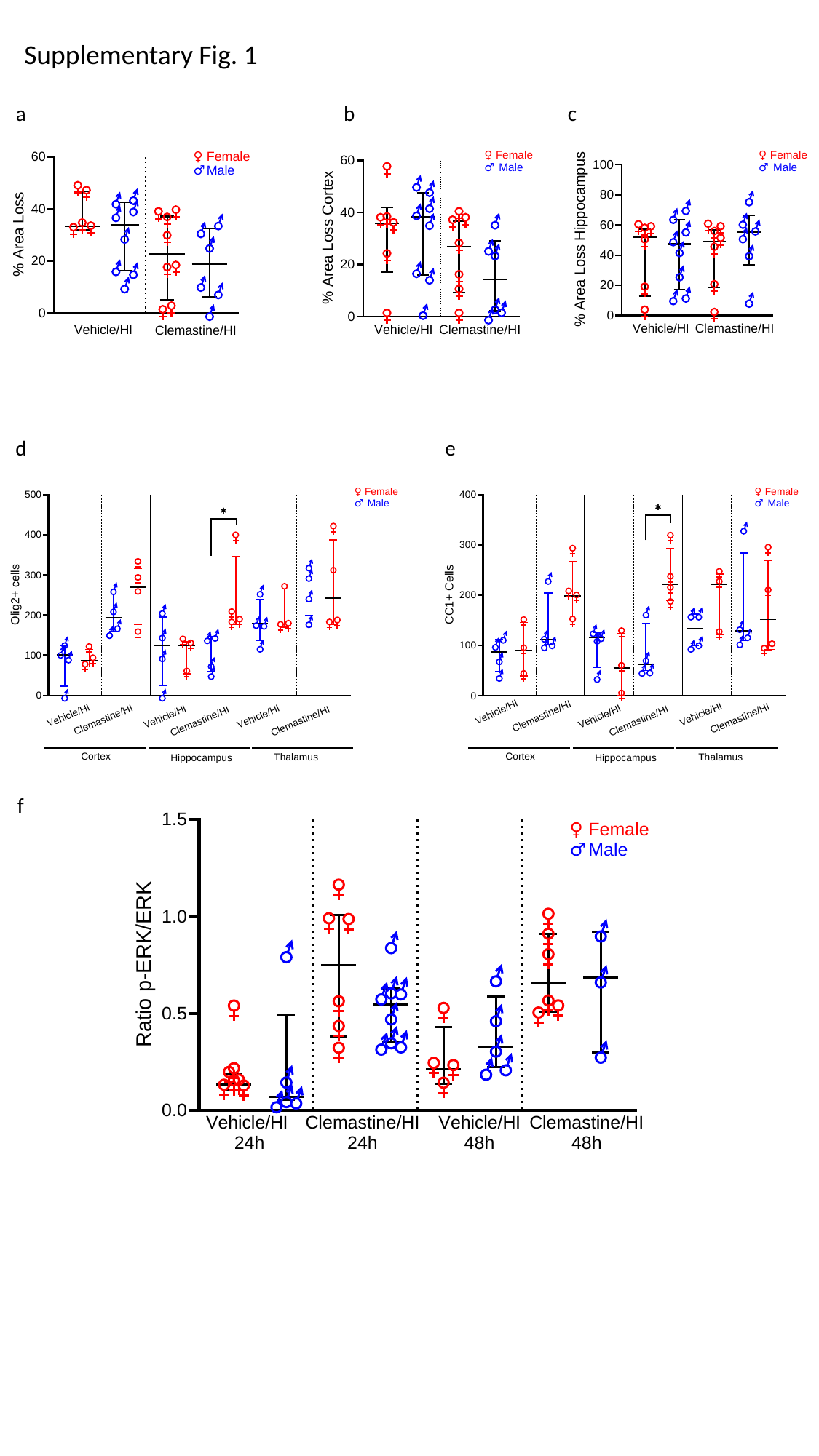

Supplementary Fig. 1
c
b
a
e
d
f

## Slide 3
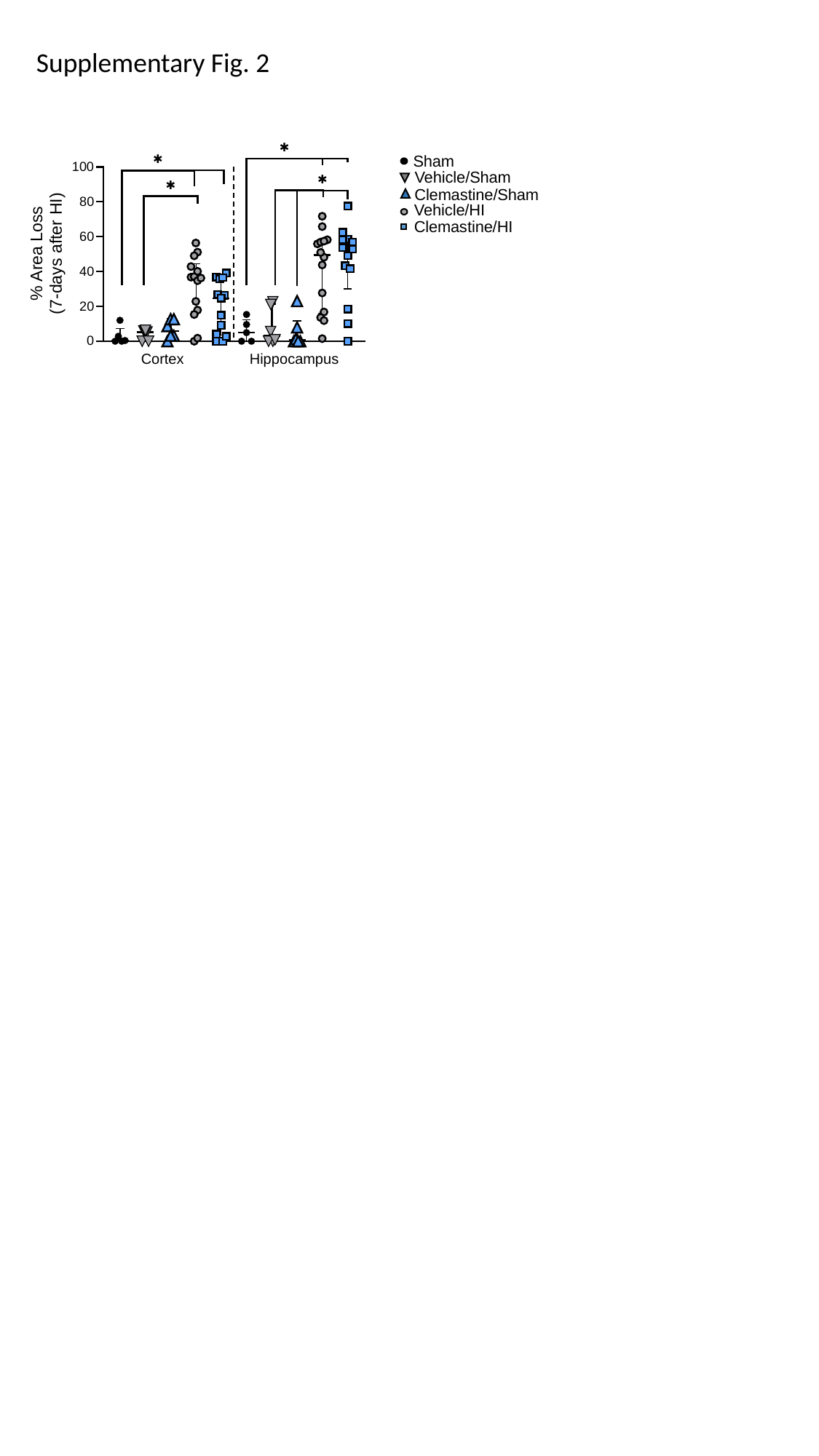

Supplementary Fig. 2
Sham
Vehicle/Sham
Clemastine/Sham
Vehicle/HI
Clemastine/HI

## Slide 4
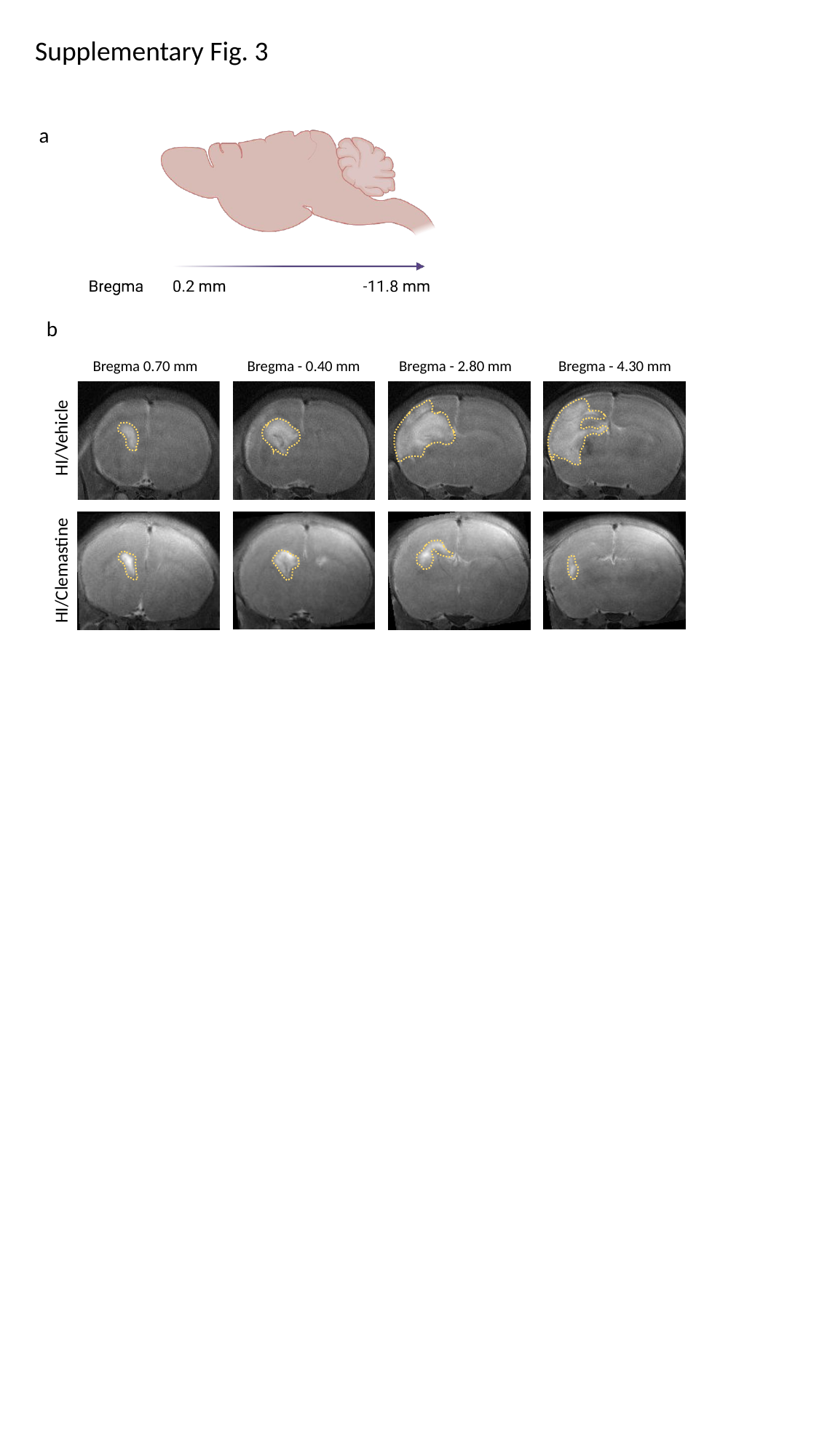

Supplementary Fig. 3
a
b
Bregma 0.70 mm
Bregma - 0.40 mm
Bregma - 2.80 mm
Bregma - 4.30 mm
HI/Vehicle
HI/Clemastine

## Slide 5
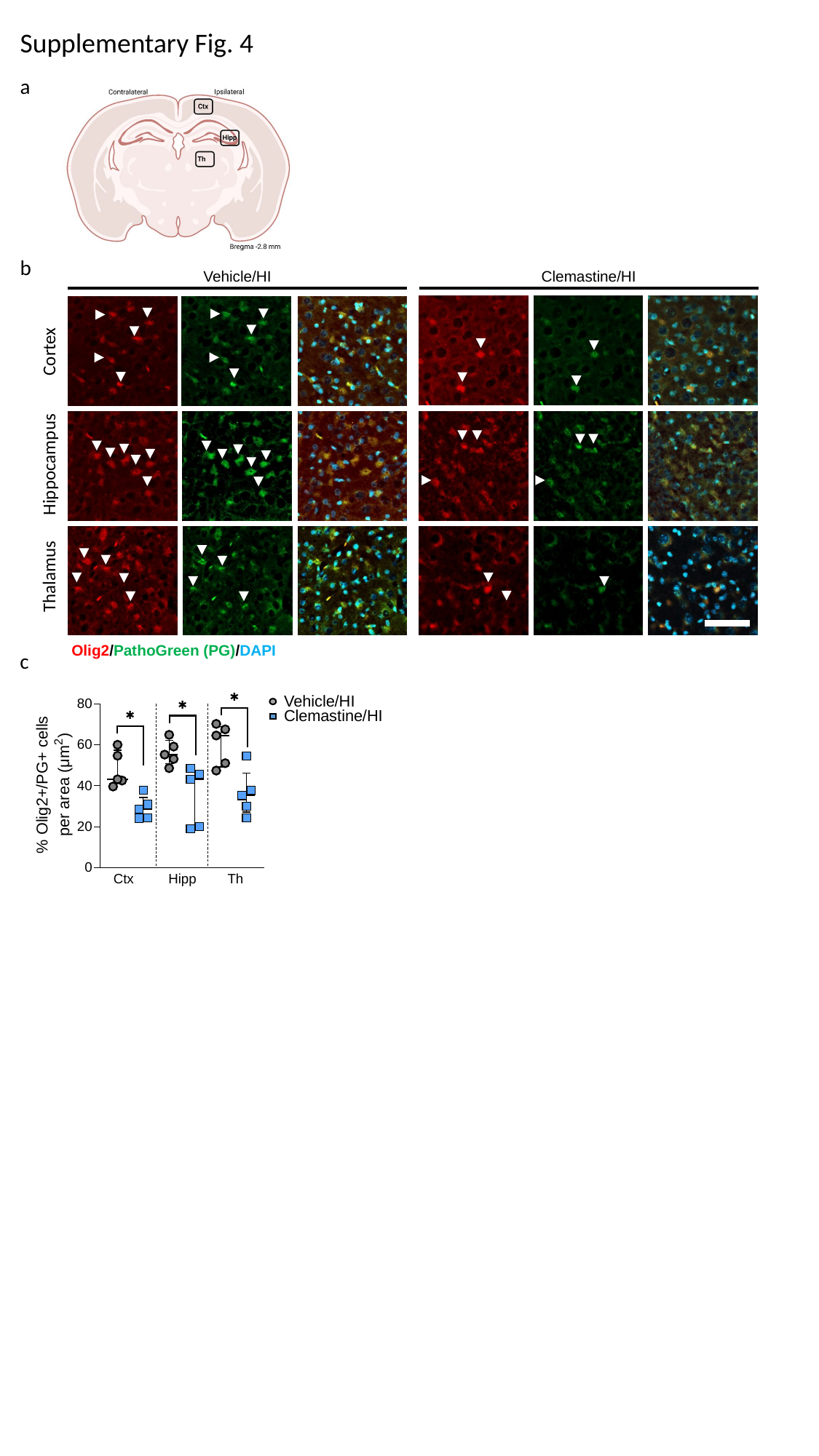

Supplementary Fig. 4
a
b
Vehicle/HI
Clemastine/HI
Cortex
Hippocampus
Thalamus
Olig2/PathoGreen (PG)/DAPI
c
Vehicle/HI
Clemastine/HI

## Slide 6
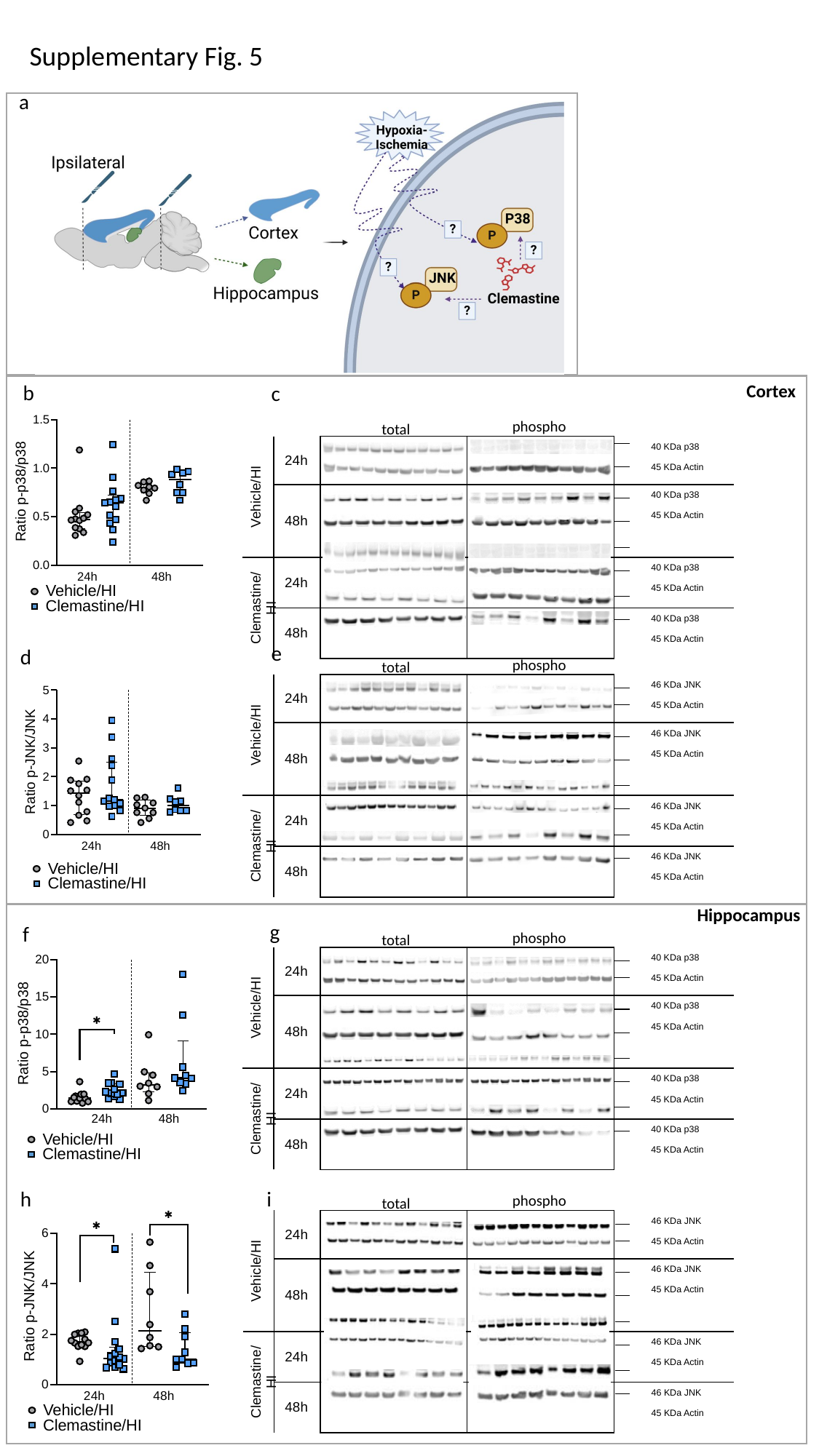

Supplementary Fig. 5
a
Cortex
b
c
phospho
total
| Vehicle/HI | 24h | | | | 40 KDa p38 45 KDa Actin |
| --- | --- | --- | --- | --- | --- |
| | 48h | | | | 40 KDa p38 45 KDa Actin |
| Clemastine/HI | 24h | | | | 40 KDa p38 45 KDa Actin |
| | 48h | | | | 40 KDa p38 45 KDa Actin |
Vehicle/HI
Clemastine/HI
e
d
phospho
total
| Vehicle/HI | 24h | | | | 46 KDa JNK 45 KDa Actin |
| --- | --- | --- | --- | --- | --- |
| | 48h | | | | 46 KDa JNK 45 KDa Actin |
| Clemastine/HI | 24h | | | | 46 KDa JNK 45 KDa Actin |
| | 48h | | | | 46 KDa JNK 45 KDa Actin |
Vehicle/HI
Clemastine/HI
Hippocampus
g
f
phospho
total
| Vehicle/HI | 24h | | | | 40 KDa p38 45 KDa Actin |
| --- | --- | --- | --- | --- | --- |
| | 48h | | | | 40 KDa p38 45 KDa Actin |
| Clemastine/HI | 24h | | | | 40 KDa p38 45 KDa Actin |
| | 48h | | | | 40 KDa p38 45 KDa Actin |
Vehicle/HI
Clemastine/HI
i
h
phospho
total
| Vehicle/HI | 24h | | | | 46 KDa JNK 45 KDa Actin |
| --- | --- | --- | --- | --- | --- |
| | 48h | | | | 46 KDa JNK 45 KDa Actin |
| Clemastine/HI | 24h | | | | 46 KDa JNK 45 KDa Actin |
| | 48h | | | | 46 KDa JNK 45 KDa Actin |
Vehicle/HI
Clemastine/HI

## Slide 7
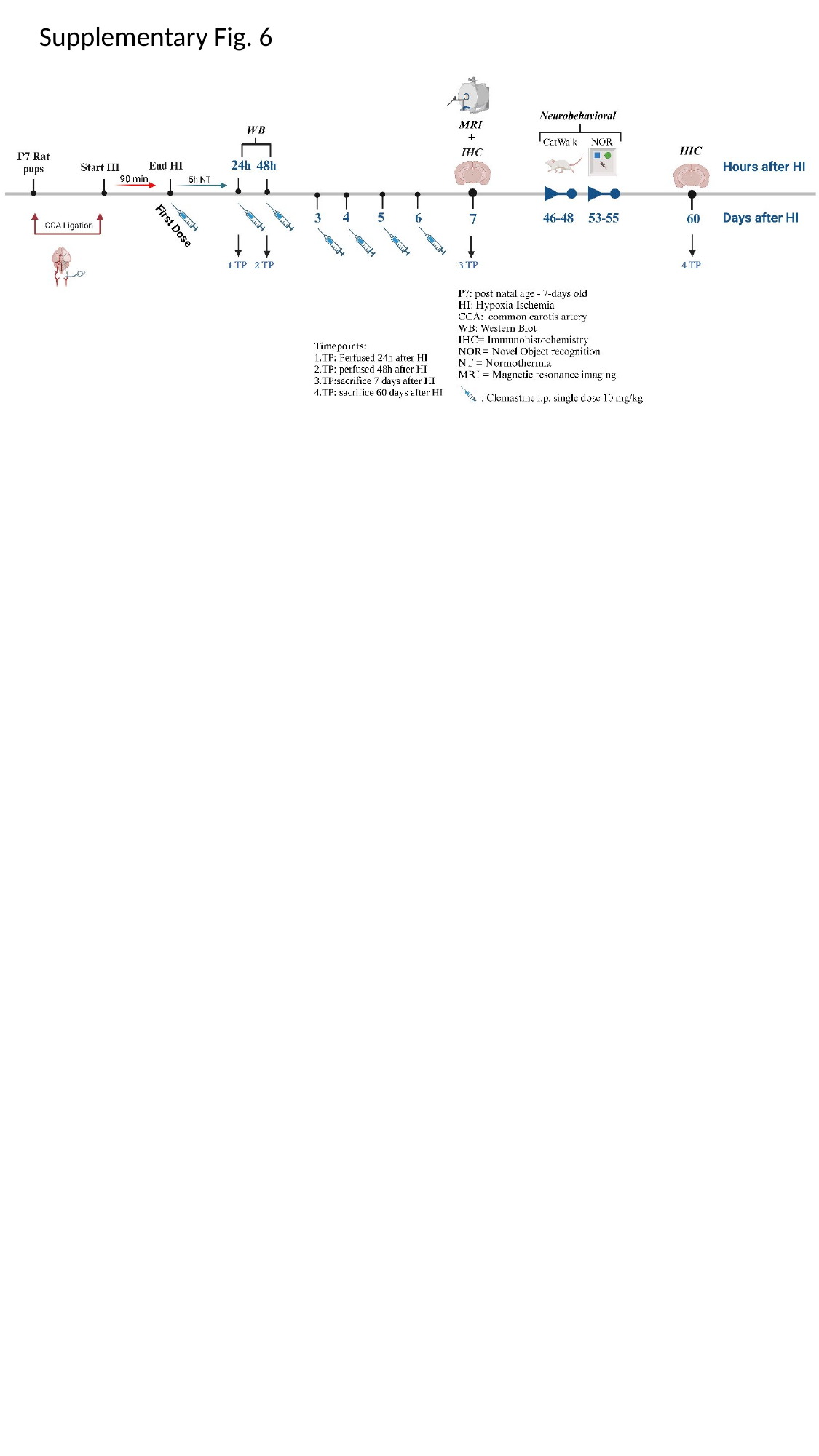

Supplementary Fig. 6
